# Continuous renal replacement therapy attenuates polymorphonuclear myeloid-derived suppressor cell expansion in pediatric severe sepsis

**DOI:** 10.3389/fimmu.2022.990522

**Published:** 2022-10-03

**Authors:** Shuyun Feng, Yun Cui, Yiping Zhou, Lujing Shao, Huijie Miao, Jiaying Dou, Tiantian Liu, Chunxia Wang, Yucai Zhang

**Affiliations:** ^1^ Department of Critical Care Medicine, Shanghai Children’s Hospital, Shanghai Jiao Tong University School of Medicine, Shanghai, China; ^2^ Institute of Pediatric Infection, Immunity, and Critical Care Medicine, Shanghai Children’s Hospital, Shanghai Jiao Tong University School of Medicine, Shanghai, China; ^3^ Institute of Pediatric Critical Care, Shanghai Jiao Tong University, Shanghai, China; ^4^ Clinical Research Unit, Shanghai Children’s Hospital, Shanghai Jiao Tong University School of Medicine, Shanghai, China

**Keywords:** pediatric intensive care units, myeloid-derived suppressor cells, continuous renal replacement therapy, sepsis, mortality

## Abstract

**Background:**

Myeloid-derived suppressor cells (MDSCs) expansion is an important mechanism underlying immunosuppression during sepsis. Though continuous renal replacement therapy (CRRT) may attenuate hyperinflammatory response in sepsis, its role in regulating MDSCs is unknown. The aim of this study was to assess the potential role of CRRT involved in sepsis-induced MDSCs expansion in pediatric sepsis.

**Method:**

The proportion of polymorphonuclear MDSCs (PMN-MDSCs) was detected before CRRT (pre-CRRT), at 24 hours after CRRT (CRRT 1^st^ day) and on the 7^th^ day after CRRT (CRRT 7^th^ day). The correlation analyses were performed to elucidate the relationship of MDSCs with clinical indexes in sepsis.

**Results:**

Totally 22 pediatric patients with sepsis were enrolled [median age 44 (*IQR*15, 83) months]. PMN-MDSCs were expanded in pediatric sepsis compared with healthy controls (4.30% *vs*. 0.37%, *P*=0.04). The proportion of PMN-MDSCs showed a decreased tendency on the CRRT 7^th^ day compared with that on the CRRT 1^st^ day in survivors (2.29% *vs.*5.32%, *P* = 0.088). There was no significant difference in the proportion of PMN-MDSCs between survivors and non-survivors before CRRT (4.51% vs. 3.33%, *P*=0.745). The levels of interleukin 6 (IL-6) was decreased on the CRRT 7^th^ day compared with CRRT 1^st^ day in survivors. In the subgroups of patients with significantly decreased IL-6 levels after CRRT, the proportion of PMN-MDSCs on the CRRT 7^th^ day were also significantly decreased compared with that on the CRRT 1^st^ day (2.21% *vs.* 6.67%, *P* = 0.033).

**Conclusion:**

The proportion of PMN-MDSCs was down-regulated on the CRRT 7^th^ day in survivors with sepsis. The reduced PMN-MDSCs expansion may relate to decreased IL-6 level.

## Introduction

Sepsis is defined as a life-threatening organ dysfunction caused by a dysregulated host response to infection (1). The case-fatality rate of pediatric severe sepsis was still high (2). Prompt diagnosis and administration of antibiotics and supportive therapeutic options can lead to better outcomes. In 2020, Surviving Sepsis Campaign (SSC) international guidelines for sepsis-associated organ dysfunction in children suggested that continuous renal replacement therapy (CRRT) should be considered in patients for facilitating management of fluid overload and acute kidney injury (AKI) (3). It is well-known that CRRT clears endotoxins, inflammatory mediators and removes excess fluids (4), consequently to maintain hemodynamic stability or potentially contribute to improving hyperinflammatory response (5). Our previous studies indicated that serum IL-6 and TNF-α levels were significantly decreased in patients with sepsis or secondary hemophagocytic lymphohistiocytosis/macrophage activation syndrome received CRRT (6, 7). However, the potential role of CRRT involved in regulation of immune hemostasis is largely unknown.

Growing interest focuses on a heterogeneous population of cells from the myeloid lineage, called myeloid-derived suppressor cells (MDSCs), which have been shown to exert potent immunosuppression in inflammatory diseases and cancer (8, 9). Usually, two major subpopulations of MDSCs are generally considered: polymorphonuclear MDSCs (PMN-MDSCs, previously known as granulocyte-MDSCs [G-MDSCs]) and monocytic MDSCs (M-MDSCs). MDSC inhibits Th1 responses and induces Th2 responses and regulatory T (Treg) cells through the production of arginase-1(ARG-1), inducible NO-synthase (iNOS), and reactive oxygen species (ROS) (10). L-arginine depletion which is sustained by the production by MDSCs of ARG-1 also affects the function of T cells (11). The inflammatory environment stimulates the recruitment of immature myeloid cells from the bone marrow into the blood stream, leading to immunosuppressive functions during sepsis (12). MDSCs are immature myeloid cells that normally make up about 0.5% of peripheral blood mononuclear cells (PBMCs), but the proportion of MDSCs is as high as 10% in septic patients (13). Mathias et al. (14) found that higher circulating MDSCs are independently associated with poor long-term outcomes in sepsis. Therefore, MDSCs may become an interesting therapeutic target in severe sepsis and septic shock.

Until now, whether CRRT regulates the proportion of MDSCs or improves immunosuppression has not been reported in sepsis. In the present study, we detected the proportion of MDSCs in children with severe sepsis received CRRT, and analyzed the potential roles of CRRT in regulation of MDSCs expansion during sepsis.

## Materials and methods

### Study design and population

**Figure 1:**
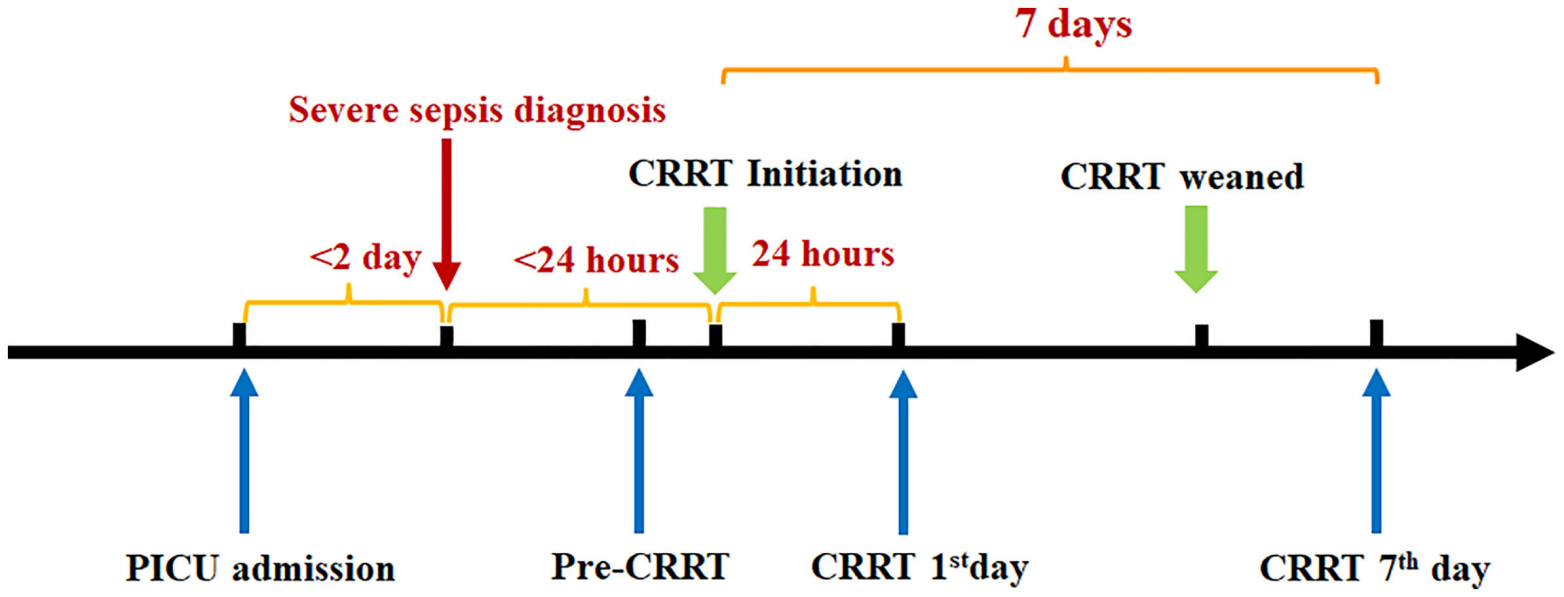
The schematic diagram of study design.

An *ex vivo* pilot study based on small simples was conducted in the pediatric intensive care unit (PICU) at Shanghai Children’s Hospital, Shanghai Jiao Tong University School of Medicine. Patients with severe sepsis (within 2 days after PICU admission) received CRRT (within 24 hours after sepsis diagnosis) were enrolled from January 2020 to December 2021. The schematic diagram of study design was shown in **Figure 1**. The inclusion criteria were as follows (1): aged over 28 days to 18 years old (2); diagnosed with severe sepsis according to the International Pediatric Sepsis Consensus Conference in 2005 (15) (3); the length of PICU stay for over 24 hours. The exclusion criteria included (1): patients with heredity metabolic disease (2); patients with advanced tumor. For patients who had repeated admissions to PICU, only the first PICU admission was included for analysis. The research protocol conducted in accordance with the ethical guidelines of the 1975 Declaration of Helsinki was approved by the Ethics Committee of Shanghai Children’s Hospital (2018R039-E02,2018R039-E03,2018R039-E04). The informed consent to CRRT or enrollment into this study was provided by the participants’ parents or relatives.

### Blood samples

Patients enrolled in study were diagnosed with severe sepsis within 2 days of PICU admission and received CRRT within 24 hours of diagnosis. Blood samples was collected in sterile vacuum blood collection tubes between severe sepsis diagnosed and CRRT initiation as the pre-CRRT, then collected at 24 hours after CRRT (CRRT 1^st^ day) on the 7^th^ day after CRRT (CRRT 7^th^ day). The schematic diagram of study design was shown in **Figure 1**. The residual blood from healthy children for physical health check were used as control (n=15). PBMCs were isolated by Ficoll density gradient (Dakewe Biotech Co.,Ltd. Cat:721101). Then, the proportions of MDSCs were analyzed using flow cytometry. The delay between sampling and beginning of flow cytometry was <1 hour.

### Flow cytometry

PBMCs were obtained from fresh heparinized human blood after Ficoll density centrifugation. Red blood cells were lysed with lysis buffer (Cat:349202, BD Biosciences, San Jose, CA, USA) for 10 min at room temperature and the remaining cells were washed twice with PBS. The remaining cells were stained for 10 min at room temperature in dark with the following antibodies: PE-A-labeled anti-human HLA-DR, APC-A-labeled anti-human CD33, FITC-A- labeled anti-human CD15, APC-H7-A-labeled anti-human CD11b, and V450-A-labeled anti-human CD14 (Beckman Coulter, Inc. REF:IM1639, IM2471, B36298; Biolegend, Inc. Cat:101226,325628). Red blood cells were then lysed with lysis buffer for 10 min and the remaining cells were washed twice with PBS. Following by two wash steps, cell acquisition was performed using Navios flow cytometer and the data were analyzed by FlowJo 10.5.3. Gating strategy was displayed in results.

### Data collection

Medical records were reviewed to extract the demographic data (such as age, gender), clinical features, the length of PICU stay, and pediatric Sequential Organ Failure Asses score (p-SOFA) on PICU admission. Laboratory parameters included white blood cells (WBC), platelet (PLT), C-reactive protein(CRP), procalcitonin (PCT);and indicators for acute liver injury such as total bilirubin (TBIL), alanine aminotransferase (ALT), γ-Gglutamyltransferase (γ-GT), lactate dehydrogenase (LDH); coagulation indicators such as prothrombin time (PT), anginal partial thromboplastin time (APTT), international normalized ratio (INR), fibrinogen (Fib), D-dimer; and indicators for acute kidney injury such as creatinine (Cr), and blood urea nitrogen (BUN)); and immune-related indicators: the percentage of peripheral blood T-cell subsets (CD4^+^ and CD8^+^), natural killer cells (CD16^+^CD56^+^CD3-), B-cells (CD19^+^), cytokines (IL-6, IL-8, IL-10, TNF-α, IFN-γ); and hemodynamics indicator of serum lactate (Lac); and creatine kinase muscle/brain lsoenzyme (CK-MB). All these variables were obtained on corresponding time period as same as blood sample.

### Statistical analysis

All statistical analyses were performed by SPSS 22.0 software (IBM, Armonk, NY, USA). The continuous data were appropriately expressed as mean ± standard deviation (mean ± SD) or median (interquartile range, IQR) and analyzed using the Student’s *t* test or Mann-Whitney *U*-test, respectively. *Pearson* correlation test was used to analyze the correlation between the frequency of PMN-MDSCs and clinical or laboratory indexes. All statistical tests were two-tailed and a value of *P* < 0.05 was considered statistically significant.

## Results

### The proportion of MDSCs in pediatric patients with sepsis and healthy controls

**Figure 2:**
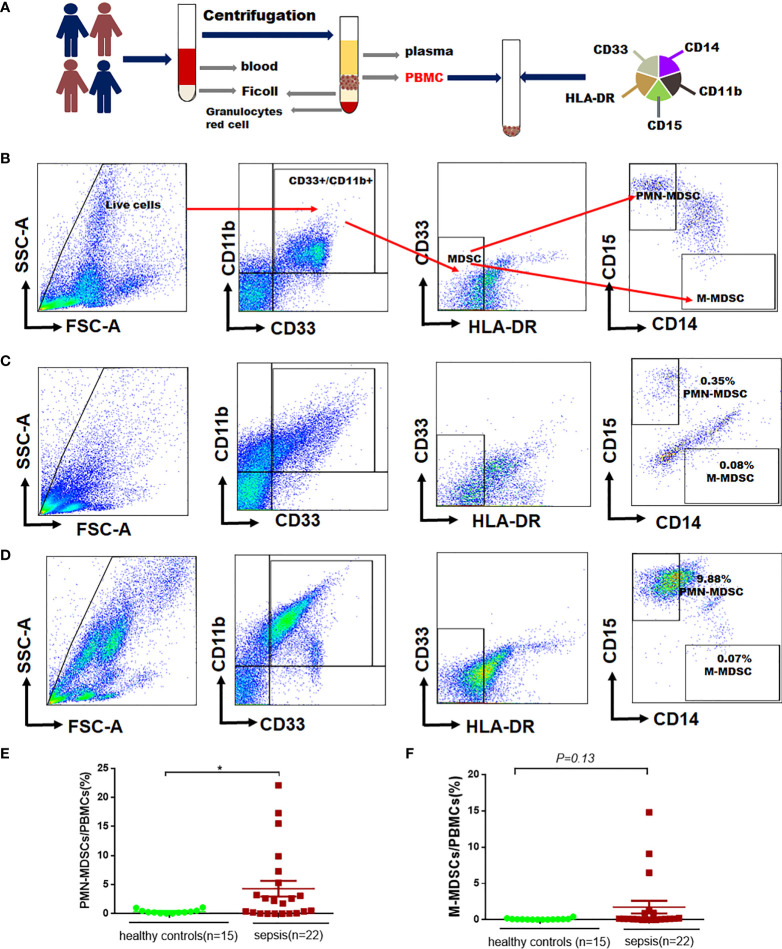
The proportion of MDSCs in PBMCs in patients with sepsis and healthy controls. **(A)** Flowchart for detecting MDSCs in peripheral blood; **(B)** Gating strategy for myeloid cells subpopulations, monocytic- (M-MDSCs) and polymorphonuclear -myeloid-derived suppressor cells (PMN-MDSCs); **(C–D)** Representative flow cytometry dot plots of M-MDSCs and PMN-MDSCs in healthy controls and patients with sepsis; **(E–F)** Percentages of PMN-MDSCs and PMN-MDSCs and M-MDSCs in PBMCs of healthy controls and patients with sepsis. * indicates *P* < 0.05.

The protocol for PBMCs isolation and MDSCs sorting were shown in **Figure 2A**. According to the reference (16), we labelled PBMCs to identify MDSCs (CD11b^+^, CD33^+^, HLA-DR^low/−^), M-MDSC (CD11b^+^, CD33^+^,CD14^+^, HLA-DR^low/−^, CD15^−^), PMN-MDSCs (CD11b^+^, CD14^−^, CD33^+^, HLA-DR^low/−^,CD15^+^) in this study (**Figure 2B**). The proportion of MDSCs in PBMCs was determined by flow cytometry in 15 healthy controls **(** **Figure 2C** **)** and 22 septic patients **(** **Figure 2D** **)**. The proportion of PMN-MDSCs in PBMCs of sepsis patients was higher than that in healthy controls (4.30% ***vs.*** 0.37%, *P*=0.04), but there was no difference in the proportion of M-MDSCs in PBMCs (1.74% ***vs.*** 0.10%, *P*=0.13) **(** **Figures 2E–F** **)**.

### Baseline characteristics of patients with sepsis received CRRT

**Figure 3:**
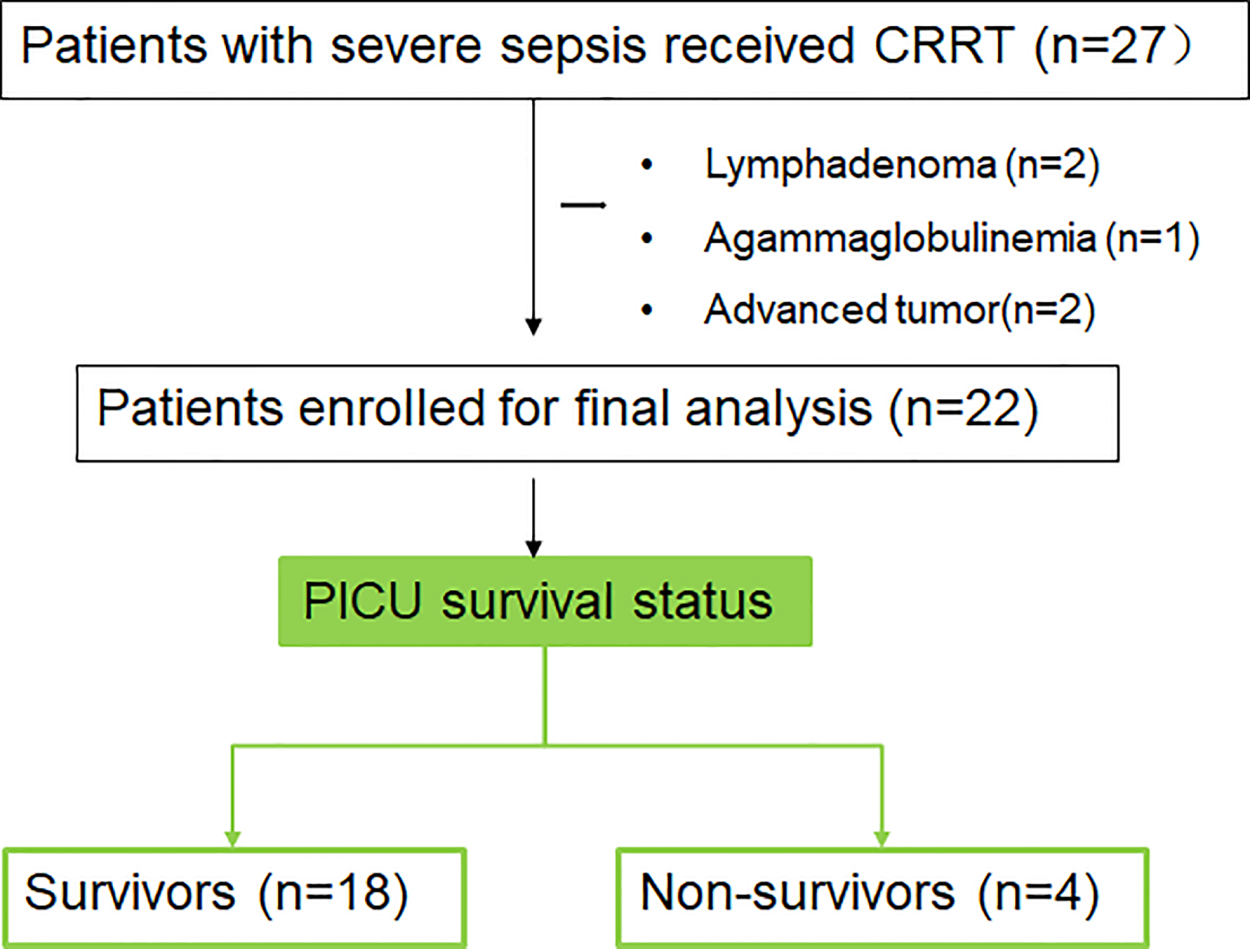
Flowchart of patients’ enrollment of this study.

A total of 27 patients diagnosed with severe sepsis received CRRT were enrolled. Patients with advanced tumor (n = 2), lymphoma (n = 2), congenital agammaglobulinemia (n = 1) were excluded. Finally, 22 subjects were enrolled in this study (**Figure 3**). According to the PICU survival status, there were 18 survivors and 4 cases were dead. The median (IQR) age was 44 (15, 83) months and 59% of patients were male. Respiratory failure was the most common reason of PICU admission (54.54%), and the respiratory tract was the most frequently site of infection (77.27%) (**Table 1**). There were no significant differences between survivors and non-survivors before CRRT in laboratory indexes including WBC, PLT, CRP, PCT, as well as indicators for organ dysfunction (**Supplementary Table 1**).

**Table 1:** Baseline characteristics of patients with severe sepsis requiring CRRT on PICU admission.

Variables	Total (n = 22)	Survivor (n = 18)	Non-survivor (n = 4)	*P value*
**Age, mo, median (IQR)**	44 (15, 83)	55 (24,83)	7 (2, 116)	0.148
**Male, n (%)**	13 (59)	11 (61)	2 (50)	>0.999
**pSOFA, median (IQR)**	7 (4, 9)	7 (4, 9)	9 (6, 10)	0.121
**Origin of infection, n (%)**				
Respiratory	17(77.27)	14 (77.78)	3 (75)	> 0.999
Gastrointestinal tract	7 (31.81)	7 (38.89)	0 (0)	0.263
Blood flow	15 (68.18)	12 (66.67)	3 (75)	>0.999
CNS	4 (18.18)	3 (16.67)	1 (25)	>0.999
Others	3 (13.63)	3 (16.67)	0 (0)	>0.999
**Reason for PICU admission, n (%)**				
Shock	10 (45.45)	8 (44.45)	2 (50)	>0.999
Acute kidney injury	1 (4.55)	0 (0)	1 (25)	0.182
Respiratory failure	12 (54.54)	8 (44.45)	4 (100)	0.096
Acute liver failure	4 (18.18)	3 (16.67)	1 (25)	>0.999
Gastrointestinal dysfunction	6 (27.27)	5 (27.78)	1 (25)	>0.999
Encephalopathy	4 (18.18)	3 (16.67)	1 (25)	>0.999

IQR, interquartile range; pSOFA, pediatric Sequential Organ Failure Asses score; PRISMIII, pediatric risk of mortality III; CNS, central nervous system.

### The proportion of MDSCs in PBMCs of patients received CRRT

**Figure 4:**
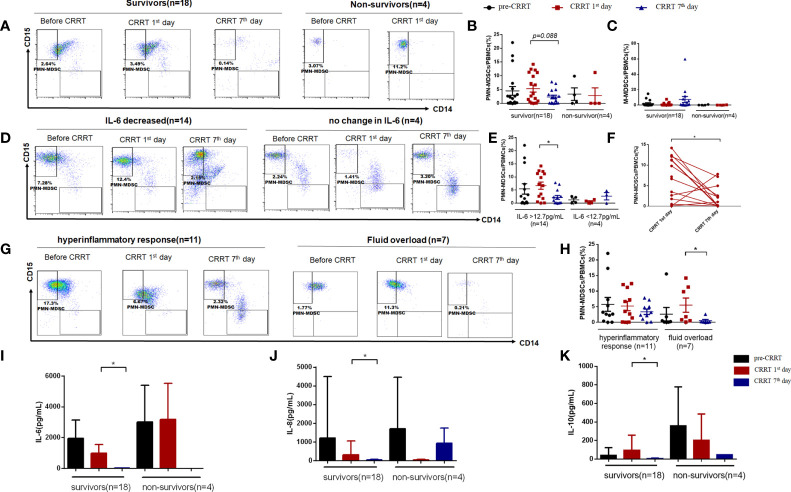
The proportion of MDSCs in PBMCs of patients with severe sepsis who received CRRT. **(A)** Representative flow cytometry dot plots of PMN-MDSCs (CD11b^+^, CD14^−^, CD33^+^, HLA-DR^low/−^,CD15^+^) in survivors and non-survivors; **(B, C)** the percentage of PMN-MDSCs and M-MDSCs in PBMCs in survivors and non-survivors; **(D, E)** Representative flow cytometry dot plots of PMN-MDSCs in children with decreased IL-6 levels and unchanged IL-6 levels **(D)**, as well as the median proportion of PMN-MDSCs in PBMCs in these patients **(E)**; **(F)** The proportion of PMN-MDSCs in each patient with decreased serum IL-6 levels after CRRT (n = 14); **(G, j, H)** Representative flow cytometry dot plots of PMN-MDSCs in patients received CRRT for hyperinflammatory response (n = 11) or fluid overload (n = 7), respectively **(G)**, as well as the median proportion of PMN-MDSCs in PBMCs in these patients **(H)**; **(I–K)** Serum levels of IL-6 **(I)**, IL-8 **(J)**, and IL-10 **(K)** before CRRT and on the CRRT 1^st^ day and 7^th^ day in survivors (n = 18) and non-survivors (n = 4). * indicates *P* < 0.05.

Given that the percentage of MDSCs in PBMCs of children with sepsis were higher than that of healthy children, we divided the children into different groups for further analysis. There was no significant difference in the proportion of PMN-MDSCs between survivors and non-survivors before CRRT (4.51% *vs.* 3.33%, *P=0.745*). The proportion of PMN-MDSCs on the CRRT 7^th^ day showed a decreased tendency compared CRRT 1^st^ day in survivors (2.29% vs. 5.32%, *P*=0.088) (**Figures 4A, B**), while the percentage of M-MDSCs in PBMCs did not change obviously in each time period (**Figure 4C**). According to the serum IL-6 levels on the 7^th^ day after CRRT, survivors were divided into decreased IL-6 group (n = 14) and non-decreased IL-6 group (n = 4) (decreased IL-6 group was defined as the patient who with over normal level of IL-6 [>12.7pg/mL] before CRRT initiation, then serum IL-6 level was significantly decreased after CRRT). In patients with decreased IL-6 levels, the frequency of PMN-MDSCs in PBMCs on the CRRT 7^th^ day decreased significantly compared with CRRT 1^st^ day (2.21% vs. 6.67%, *P*=0.033) (**Figures 4D, E**). Furthermore, the percentage of PMN-MDSCs in PBMCs showed significant decreased at on the CRRT 7^th^ day compared with CRRT 1^st^ day among patients with decreased IL-6 levels (n = 14, **Figure 4F**). Moreover, based on the indications for CRRT (for hyperinflammatory response [n = 11] or fluid overload [n = 7]), the proportion of PMN-MDSCs in PBMCs decreased obviously on the CRRT 7^th^ day compared with CRRT 1^st^ day (0.51% vs. 5.50%, *P*=0.028) (**Figures 4G, H**). At the same time, the levels of IL-6, IL-8, and IL-10 in serum were significantly decreased on the CRRT 7^th^ day compared with CRRT 1^st^ day in survivors (10.70pg/mL vs. 987.62 pg/mL, *P*=0.002; 30.69 pg/mL vs. 320.37 pg/mL, *P*=0.004; 4.73 pg/mL vs. 93.50 pg/mL, *P*=0.023) (**Figures 4I–K**).

### Correlations of clinical indexes and the proportion of MDSCs

**Figure 5:**
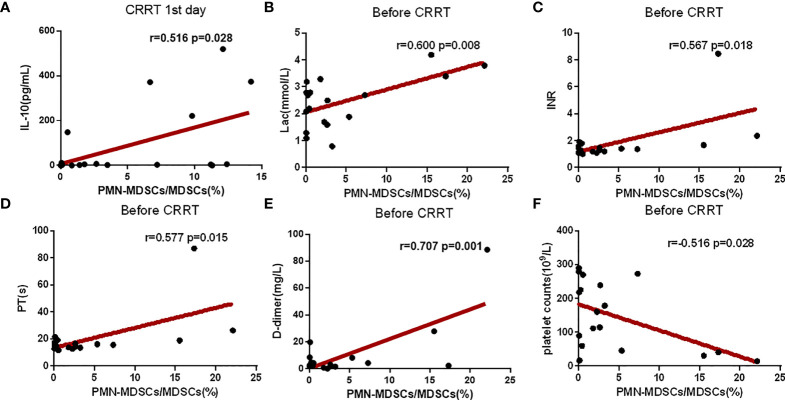
Correlation analysis of clinical indexes and the proportion of PMN-MDSC in PBMCs. **(A–F)** Correlation analysis between the proportion of PMN-MDSC in PBMCs and IL-10, Lac, platelet count, INR, prothrombin time, and D-dimer. The time points for collection data of PMN-MDSCs and clinical indicators were matched on the CRRT 1^st^ day **(A)** or before CRRT **(B–F)**.

The proportion of PMN-MDSC in PBMCs was positively correlated with serum IL-10 on the CRRT 1^st^ day (**Figure 5A**). Before CRRT, the proportion of PMN-MDSC in PBMCs was positively correlated with lactate levels (**Figure 5B**), as well as coagulation-related indicators, such as INR, prothrombin time and D-dimer (**Figures 5C–E**). In addition, the proportion of PMN-MDSC in PBMCs was negatively correlated with the number of platelets (**Figure 5F**). In another aspect, the frequency of CD8^+^ on the CRRT 7^th^ day was significantly increased compared with CRRT 1^st^ day, the levels of lymphocytes were increased with trend but without statistical significance; in addition, the levels of IL-6 were significantly decreased on the CRRT 7^th^ day compared with CRRT 1^st^ day (**Table 2**).

**Table 2:** The changes of the percentages of immune cells in pediatric patients with severe sepsis received CRRT.

Parameters	Before CRRT	CRRT 1^st^ day	CRRT 7^th^ day	*P* value (before vs. 1^st^ day)	*P* value (24h vs.7^th^ day)
CD8^+^, %	17.45 (13.80, 30.58)	20.42 (15.66,23.71)	26.51 (20.00, 35.40)	0.878	0.025*
CD4^+^, %	33.92 (26.84, 45.87)	28.6 (20.76, 39.54)	31.13 (25.98, 49.93)	0.169	0.208
CD4^+^/CD8^+^, %	1.84 (1.40, 2.25)	1.38 (0.98, 1.87)	1.28 (0.98, 1.86)	0.114	0.726
CD19^+^, %	32.68 (13.49, 49.58)	38.28 (19.83, 48.80)	28.07 (13.96, 36.89)	0.374	0.176
CD16^+^CD56^+^/CD3^-^, %	5.87 (3.54, 9.74)	2.64 (1.33, 5.92)	4.43 (1.84, 9.64)	0.069	0.398
Lymphocytes, 10^9^/L	0.75 (0.45, 1.54)	1.24 (0.70, 2.81)	2.099 (1.37, 3.80)	0.074	0.093
IL-6, pg/mL	147.21 (7.82, 3985.73)	31.75 (11.68, 1019.75)	3.97 (0.29, 23.26)	0.114	0.002*

* indicates P < 0.05.

### Detection of indexes for immunological function in patients with severe sepsis received CRRT

**Figure 6:**
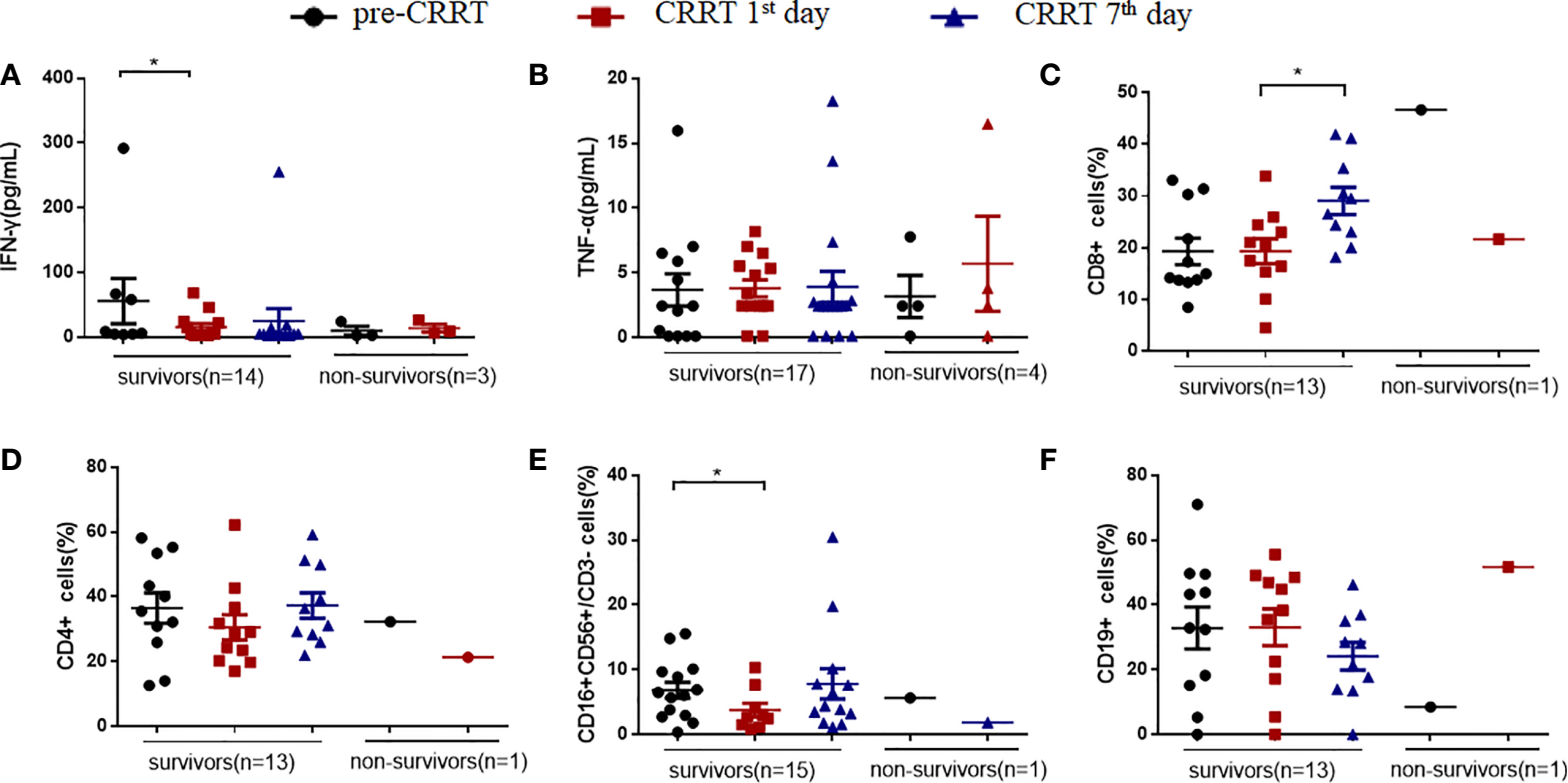
The percentage of immune cells and the levels of cytokines in patients with sepsis received CRRT. **(A, B)** levels of interferon γ (IFN-γ) and tumor necrosis-α (TNF-α); **(C–F)** the percentages of CD8^+^ T cells **(C)**, CD4^+^ T cells **(D)**, NK(CD16^+^CD56^+^CD3-) cells **(E)**, CD19^+^ B cells **(F)**. * indicates P<0.05.

MDSCs-induced immunosuppression is related to the suppressed T cells proliferation (13, 17). The secretion of interferon γ (IFN-γ) in the survivors showed a decreased tendency on the CRRT 1^st^ day compared with that at pre-CRRT (15.57pg/mL vs. 55.70 pg/mL, *P*=0.018, **Figure 6A**), while there was no significant difference in serum TNF-α **(** **Figure 6B** **)**. On the CRRT 7^th^ day, the percentage of CD8+ T cells in survivors was significantly higher than that on the CRRT 1^st^ day (29.06% *vs.* 19.15%, *P*=0.025, **Figure 6C**), while the percentage of CD4+ T cells did not change significantly **(** **Figure 6D** **)**. The percentage of NK cells decreased on the CRRT 1^st^ day compared with pre-CRRT (3.75% *vs.* 6.84%, *P*=0.023, **Figure 6E**), while B(CD19+) cells did not change significantly during the progress of sepsis **(** **Figure 6F** **)**.

## Discussion

MDSCs expansion is one of main causes for sepsis-induced immunosuppression related to organ dysfunction and worse outcome. In this study, we demonstrated that PMN-MDSCs were expanded in pediatric sepsis. To the best of our knowledge, it is the first report that the proportion of PMN-MDSCs was reduced after CRRT in survivors, associated with decreased serum IL-6 levels. Moreover, there was significant recovery of CD8+ cells proliferation on the CRRT 7^th^ day in survivors. These results suggest that CRRT reduces MDSCs expansion possibly contributed by clearing IL-6, consequently leading to clinical benefits in patients with sepsis.

Mathias B et al. (14) reported that the proportion of MDSCs in white blood cells reaches up to 45% in patients with severe sepsis/septic shock, and a high percentage of MDSCs is associated with early mortality, a longer hospital stays, and nosocomial infections. Both M-MDSCs and PMN-MDSCs significantly contribute to T-cell dysfunction in septic patients, and PMN-MDSCs appear to be a major actor of sepsis-induced immunosuppression (18). In our study, the proportion of PMN-MDSCs in PBMCs, but not M-MDSCs, showed a decreased tendency on the 7^th^ day after CRRT compared with on the 1^st^ day after CRRT in survivors.

Our previous study indicated that CRRT significantly decreases serum levels of TNF-α and IL-6 in sepsis (19, 20). Several evidences identified that the expansion and activation of MDSCs were dependent on IL-6 and NF-κB signaling pathways (21, 22). Interestingly, we found that the proportion of PMN-MDSCs decreased significantly on the CRRT 7^th^ day in patients with decreased serum IL-6 levels after CRRT. It has been demonstrated that IL-6 contributes to the accumulation and activation of MDSC in cancer (8). Targeting IL-6 or IL-6 receptor (IL-6R) for tumor immunotherapy to block MDSC-mediated immunosuppression in cancer patients (23). The results suggest that the potential relationship of significantly decreased IL-6 levels lead to down-regulated PMN-MDSCs expansion by CRRT.

Patients with sepsis receive CRRT due to indications including control of hyperinflammatory response and fluid overload, as well as AKI. The proportion of PMN-MDSCs in PBMCs decreased obviously in patients who received CRRT suggesting a possible association between MDSCs and hemodynamic homeostasis. Kandarp H S et al. (24) found that MDSCs suppressed the increase of blood pressure *via* the production of hydrogen peroxide and depletion of hypertensive MDSCs increased blood pressure and renal inflammation. Furthermore, augmenting MDSCs reduces the detrimental cardiovascular and renal effects of cyclosporine A in mice and may be a therapeutic strategy for cyclosporine A-treated patients (25). Whether CRRT improving hemodynamic disorder contributes to the reduced levels of PMN-MDSCs needs more attention in the future.

MDSCs suppress the proliferation of CD8+ cells and promote the secretion of IFN-γ, leading to immunosuppression (17, 26). Moreover, MDSCs directly inhibit the anti-tumor functions of T cells and NK cells (22). In the present study, we found that the proportion of CD8+ cells displayed an increased recovery on the CRRT 7^th^ day, suggesting that there was possible relationship between CRRT-reduced MDSCs levels and T cell activation. Numerous studies have reported the relationship between IL-6, IL-8, IL-10 and MDSCs. In patient with heart failure, both the percentage of human leukocyte MDSCs in the blood and plasma level of IL-6, IL-10 were significantly increased (27). In addition, IL-6 and IL-8 are linked with MDSCs accumulation and correlate with poor clinical outcomes in melanoma patients (28). In the present study, we found that the levels of IL-6 in serum were significantly decreased on the CRRT 7^th^ day compared with the levels on the CRRT 1^st^ day, and the proportion of MDSCs in PBMCs during this time period also decreased significantly. Correlation analysis showed that the level of IL-10 in serum was positively correlated with the proportion of MDSCs, because IL-10 drives the signaling pathway that generates MDSCs and enhances immunosuppression during late sepsis, and blocking IL-10 prevents MDSC expansion during late sepsis (29). The underlying mechanisms of IL-6 and IL-10 should be further investigated.

Correlation analysis also showed that serum lactate levels, platelet counts were positively correlated with the proportion of MDSCs in PBMCs of sepsis patients. Yang et al. demonstrated that lactate derived from irradiated tumor cells mediated MDSC activation (30), and the lactate metabolism regulates MDSC development through the Hes1/MCT2/c-Jun axis (31). In addition to the association between MDSCs and lactate, there is also an association between MDSCs and platelets. Expansion of PMN-MDSC contributes to platelet activation by L-Arginine deprivation during SARS-CoV-2 infection (32). In addition, the percentage of PMN-MDSCs in PBMCs also was correlated with PT, INR, and D-dimer. The correlation between MDSCs and coagulation indexes has also been reported in other diseases. In alcoholic liver cirrhosis patients, level of plasma ARG-1 secreted by MDSCs were correlated with INR (33). In adult acute myeloid leukemia patients, MDSCs were significantly increased, and the level of MDSCs was correlated well with plasma D-dimer levels (34). Moreover, MDSCs count was correlated with PT in dengue fever patients (35). This suggests that PMN-MDSCs may affect the pathogenesis of sepsis by affecting coagulation in patients with CRRT support.

There are several limitations in this study. Firstly, our conclusions were based on a single center exploratory study with a small number of patients. So, the conclusion of this study should be further strengthened in a well-designed study with a larger population. Secondly, it was lack of the data about the classical indicators of MDSC such as ARG-1, iNOS due to the limited sample volume in children. Thirdly, patients with sepsis without CRRT support were not enrolled in this study. Nevertheless, to the best of our knowledge, it is the first pilot study to reveal the relationship between MDSCs expansion and CRRT, and these results lead to a new perspective for the potential role of CRRT in sepsis.

## Conclusion

The proportion of PMN-MDSCs in PBMCs is high at early stage of pediatric sepsis, which were significantly reduced on the 7^th^ day after CRRT in patients with severe sepsis. Further analysis implied that CRRT-reduced PMN-MDSCs might be associated with the decreased IL-6 or improved hemodynamic hemostasis. Given that PMN-MDSC is involved in sepsis-induced immunosuppression and its association with the outcome of sepsis, we suspected that the clinical benefits of CRRT-reduced PMN-MDSCs could be related to the improvement of outcome of sepsis.

## Data availability statement

The datasets presented in this study can be found in online repositories. The names of the repository/repositories and accession number(s) can be found in the article/**Supplementary Material**.

## Ethics statement

The studies involving human participants were reviewed and approved by Ethics Committee of Shanghai Children’s Hospital. Written informed consent to participate in this study was provided by the participants’ legal guardian/next of kin.

## Author contributions

YZhang and CW conceived the study, designed the trial, and obtained research funding. SF and YC performed the experiments. YC, YZhou, LS, HM, JD, and TL performed the data collection. SF analyzed the data. SF and CW drafted the manuscript, and all authors contributed substantially to its revision. YZhang takes responsibility for the paper as a whole. All authors contributed to the article and approved the submitted version.

## Funding

This study was sponsored by Science and Technology Commission of Shanghai Municipality (18411951000), Natural Science Foundation of Shanghai (20ZR1446500), and the Shanghai Municipal Health Commission (20194Y0448). CW was also supported by the Talents Program of Shanghai Municipal Education Commission-Gaofeng Clinical Medicine Grant (20171928), National Natural Science Foundation of China (82171729).

## Conflict of interest

The authors declare that the research was conducted in the absence of any commercial or financial relationships that could be construed as a potential conflict of interest.

## Publisher’s note

All claims expressed in this article are solely those of the authors and do not necessarily represent those of their affiliated organizations, or those of the publisher, the editors and the reviewers. Any product that may be evaluated in this article, or claim that may be made by its manufacturer, is not guaranteed or endorsed by the publisher.
