# Supplementary material for: Continuous renal replacement therapy attenuates polymorphonuclear myeloid-derived suppressor cell expansion in pediatric severe sepsis
Source: Front Immunol. 2022 Oct 3;13:990522. doi: 10.3389/fimmu.2022.990522 (PMC9575946; doi:10.3389/fimmu.2022.990522)
Supplement: Supplementary file 2 [file Table_1.docx]

Supplementary Material

**Supplementary Tables**

**Supplementary Table 1.** The laboratory indexes in pediatric patients with sepsis before CRRT

| **Characteristics** | **Total** | **Survivor** | **Non-survivor** | ***P value*** |
| --- | --- | --- | --- | --- |
|  | **(n = 22)** | **(n = 18)** | **(n = 4)** |  |
| **WBC, ×10^9^ /L** | 7.62 (3.47,13.38) | 7.84 (4.41,14.56) | 4.51 (1.95,10.41) | 0.179 |
| **PLT, ×10^9^ /L** | 138 (44.75,271.75) | 138 (44.75,247.75) | 225.5 (29.75,521) | 0.61 |
| **CRP, mg/L** | 77.5 (49.75,126.75) | 82 (49.75,152) | 74.5 (39.75,109.25) | 0.632 |
| **PCT, ng/mL** | 2.24 (0.4,8.11) | 2.24 (0.26,9.71) | 4.4 (0.82,7.60) | 0.734 |
| **Indicators for acute liver injury** | | | | |
| TBIL, μmol/L | 22.97 (7.37,36.80) | 22.97 (6.667,36.80) | 24.04 (9.78,50.28) | 0.925 |
| ALT, U/L | 52 (24,60) | 53(24,60) | 51.5 (21,55.75) | 0.652 |
| γ-GT, U/L | 17 (11.5,89.5) | 15 (11,75) | 65.5 (15,257) | 0.142 |
| LDH, U/L | 371 (314,695) | 351 (240,544) | 1126.5 (412.75,1951.25) | 0.11 |
| **Indicators for coagulation** | | | | |
| PT, s | 15.8 (13.8,18.45) | 16 (13.8,19.25) | 14.35 (13.22,17.05) | 0.347 |
| APTT, s | 76 (39.35,147.85) | 85.4 (39.4,153.8) | 41.7 (35.05,79.55) | 0.244 |
| INR | 1.39(1.21,1.65) | 1.42(1.21,1.75) | 1.28 (1.15,1.51) | 0.324 |
| Fib, g/L | 2.53 (1.45,3.99) | 2.53 (1.9,3.54) | 2.93 (0.76,5.22) | 0.929 |
| D-dimer | 3.24 (2.05,8.46) | 3.24 (2.18,8.46) | 3.14 (1.16,15.36) | 0.591 |
| **Indicators for AKI** | | | | |
| Cr, μmol/L | 30 (25.00,43.00) | 32 (27,52) | 20.5 (14,38.25) | 0.089 |
| BUN, mmol/L | 4.05 (2.60,5.93) | 4.35 (2.2,6.375) | 3.75 (3.07,5.55) | 0.777 |
| **Hemodynamics** | | | | |
| Lac, mmol/L | 2.5 (1.85,3.22) | 2.6 (1.67,3.22) | 2.35 (1.97,4.6) | 0.765 |
| CK-MB, U/L | 20 (13,41) | 16 (12,30) | 31 (21,54) | 0.132 |

PRISMⅢ: Pediatric Risk of Mortality Ⅲ; CRP: C-reactive protein; WBC: white blood cell; ALT: alanine aminotransferase; TBIL: total bilirubin; γ-GT: γ-glutamyl transpeptidase; Fib: fibrinogen; APTT: activated partial thromboplastin time; INR: international normalized ratio; PT: prothrombin time; PLT: platelet; Cr: creatinine; BUN: blood urea nitrogen; Alb, albumin; Lac: lactate. p-SOFA: pediatric Sequential Organ Failure Asses; PCT: procalcitonin; CK-MB: creatine kinase muscle/brain lsoenzym; LDH: lactate dehydrogenase
